# Utilization of Complementary Medicine by Pediatric Neurology Patients and Their Families in Saudi Arabia

**DOI:** 10.7759/cureus.7960

**Published:** 2020-05-04

**Authors:** Ahmed Al-Rumayyan, Hamoud Alqarni, Bader S Almanna, Naif Althonayan, Mohammed Alhalafi, Nawaf Alomary

**Affiliations:** 1 Neurology, King Saud Bin Abdulaziz University for Health Sciences, Riyadh, SAU; 2 Medicine, King Saud Bin Abdulaziz University for Health Sciences, Riyadh, SAU

**Keywords:** complementary medicine, neurology, children, alternative medicine, quran recitation

## Abstract

Introduction

Complementary medicine (CM) consumption is a common practice worldwide. The objective of this study is to find the prevalence of parents visiting the neurology clinic who utilize CM to treat their children.

Methods

This is a cross-sectional study that was done at King Abdullah Specialized Children Hospital (KASCH), Riyadh, Saudi Arabia between 2018 and 2019. By using a self-administered questionnaire, data were collected to recognize the prevalence of using CM and to identify the commonest type or method.

Results

A total of 352 parents were given the questionnaire. The prevalence of CM usage among participant was 42%, the most common type of CM was Quran recitation at 66%, followed by herbal medicine at 30% and cautery at 26%.

Conclusion

Almost half of the parents who visited the neurology clinic at KASCH have used complementary medicine for their children, and nearly three-quarters of the parents who never used CM have thought about using it. Therefore, CM is common in the Saudi Arabian culture.

## Introduction

Complementary medicine (CM) is a phrase that describes a variety of methods such as healthcare systems, practices and the use of supplementary products to reach a treatment together with the employment of advanced medicine [1]. According to a published study, it has been shown that there is a substantial usage of complementary medicine in the treatment of many chronic diseases, especially in western countries [2]. For example, in Europe, the most commonly used complementary medical practices are acupuncture, homeopathy, manual therapy or manipulation, and phototherapy or herbal medicine [3]. In Saudi Arabia, a systematic review was done which reviewed studies about the utilization of CM. They found that the most common form of CM was praying and Quran recitation, followed by herbs, honey, dietary product, and then cupping [1].

Globally, neurological disorders have a burden of 20% due to the complexity of their treatment [4]. There are many people in our society that deal with neurological disorders by using CM whether it has an approved effect or not. In Saudi Arabia, the prevalence of dealing with CM was found to be 68%. Some studies suggest that many people used the CM after they were advised by their relatives or friends [5,6]. A study was conducted in Riyadh about the use of CM in adults with neurological diseases which revealed that cupping was the most utilized method (45.4%) followed by herbs (42.3%) [7]. From the health caregiver point of view, a study was done on the physicians’ knowledge of CM. It concluded that the patient would have a better outcome if physicians have a better knowledge of CM [8].

According to local studies, many parents reach out to complementary and alternative medicine before presenting themselves to the clinic, so it is important that pediatricians need to provide appropriate counseling regarding the use of such traditional healing [9]. Another study emphasizes that physicians themselves need to be counseled and educated about CM to improve the quality of care [10]. However, it has been noticed that there is a reluctance to start discussions about complementary medicine usage at each consultation [8]. The practice of complementary medicine is not only common in our selected population, but a study that was done in Nigeria has shown that it is also common among the majority of their participants; as a result, there must be a prompt implementation of measures and policies to endorse the appropriate use of complementary and alternative medicine [11]. Furthermore, another study was conducted in Riyadh, Saudi Arabia, in which they suggest that deep investigation is recommended regarding the consumption of complementary medicine in certain diseases [12]. To fix such issues, complementary and alternative medicine has to be appropriately legalized and controlled since many patients reach out to traditional healers [4].

This study focused on finding the prevalence of parents who use complementary medicine (CM) for their children with neurological disorders from one to fourteen years of age. In addition, it identified the most common CM used in our population. We believe this will lead to recognition not only by the physicians but also by the families of the patients about the trends of CM that are used in our society, and this, in turn, should lead to safer practice and trusted communication between the two parties.

## Materials and methods

This is a cross-sectional study that was conducted with questionnaires distributed to patients/caregivers who visited the neurology clinic at King Abdullah Specialized Children Hospital (KASCH), Riyadh to establish the prevalence of complementary medicine usage. The study included any legal guardian of a child that has a neurological disorder from 1 to 14 years of age at KASCH. The data was collected by using a structured self-developed and self-administered questionnaire in the Arabic language. If the participant was unable to complete the questionnaire, the survey was conducted by an interview. To validate the questionnaire, it was reviewed by two experts and a biostatistician.

Data were analyzed using Microsoft Excel 2016 and Social Package for Statistical Package for the Social Sciences (SPSS) version 24 (IBM Corp., Armonk, NY). The data collected with a self-administered questionnaire and was cleansed and coded in Microsoft Excel then transferred into SPSS for analysis. The reliability of the scales used in the survey was assessed using Cronbach's Alpha test. Descriptive statistics were done for all study variables, including frequencies and proportions for categorical variables, the mean and standard deviation for normally distributed numerical variables and median and interquartile range for skewed distributions. The correlations between responses in questions for scales ranged from questionable to unacceptable. The questions assessing the presence of side effects towards modern or traditional medicine were inversely correlated with other questions. In addition, the embarrassment in telling the doctor about complementary medicine was not correlated with other satisfactory questions. The deletion of these questions results in improving the reliability of scales and Cronbach alpha values.

Multivariable analysis was done using statistical tests of significance, with 95% confidence (α = 0.05). A p-value of less than 0.05 was considered to show a significant difference, while a p-value of less than 0.01 was considered highly significant. Chi-square test was done to compare categorical variables, while the Mann-Whitney U test was used to compare numerical non-parametric distributions. In addition, the multivariate analysis aimed to identify high-risk categories and common determinants in these categories.

## Results

A total of 352 participants were included in the study. The analysis of demographic data showed most participants’ children were less than 10-year-old (64%), slightly more male children (55%), and a majority of fathers (86%) as legal guardians mostly between 20- to 39-year-old (72%). Participants’ income mostly ranged between 5,000-14,999 SAR (61%). In addition, half of the respondents’ origin was in the central region (51%) of the kingdom, and the vast majority were living in cities (93%). The education of most fathers (81%) and mothers (71%) was above the secondary level, which was also significantly associated with the use of complementary medicine (p-value: 0.049 and 0.023, respectively). Further demographic data are described in Table 1.

**Table 1:** General characteristics of study participants (n = 352) SAR: Saudi Riyal

Demographic Characteristics	n	%
Age of Child		
<3 years	80	23%
4-6 years	77	22%
7-9 years	68	19%
10-12 years	73	21%
13-14 years	54	15%
Gender		
Male	195	55%
Female	157	45%
Legal Guardian		
Fathers	301	86%
Mothers	47	13%
Others	3	1%
Legal Guardian's Age		
<20 years	21	6%
20-29 years	124	36%
30-39 years	124	36%
40-49 years	52	15%
>50 years	25	8%
Father's Education		
Illiterate	9	3%
Primary	28	8%
Intermediate	31	9%
Secondary	150	43%
University and above	134	38%
Mother's Education		
Illiterate	29	8%
Primary	33	9%
Intermediate	39	11%
Secondary	102	29%
University and above	148	42%
Income (SAR)		
<5,000	58	17%
5,000-9,999	127	37%
10,000-14,999	85	24%
15,000-20,000	39	11%
>20,000	39	11%

**Figure 1:**
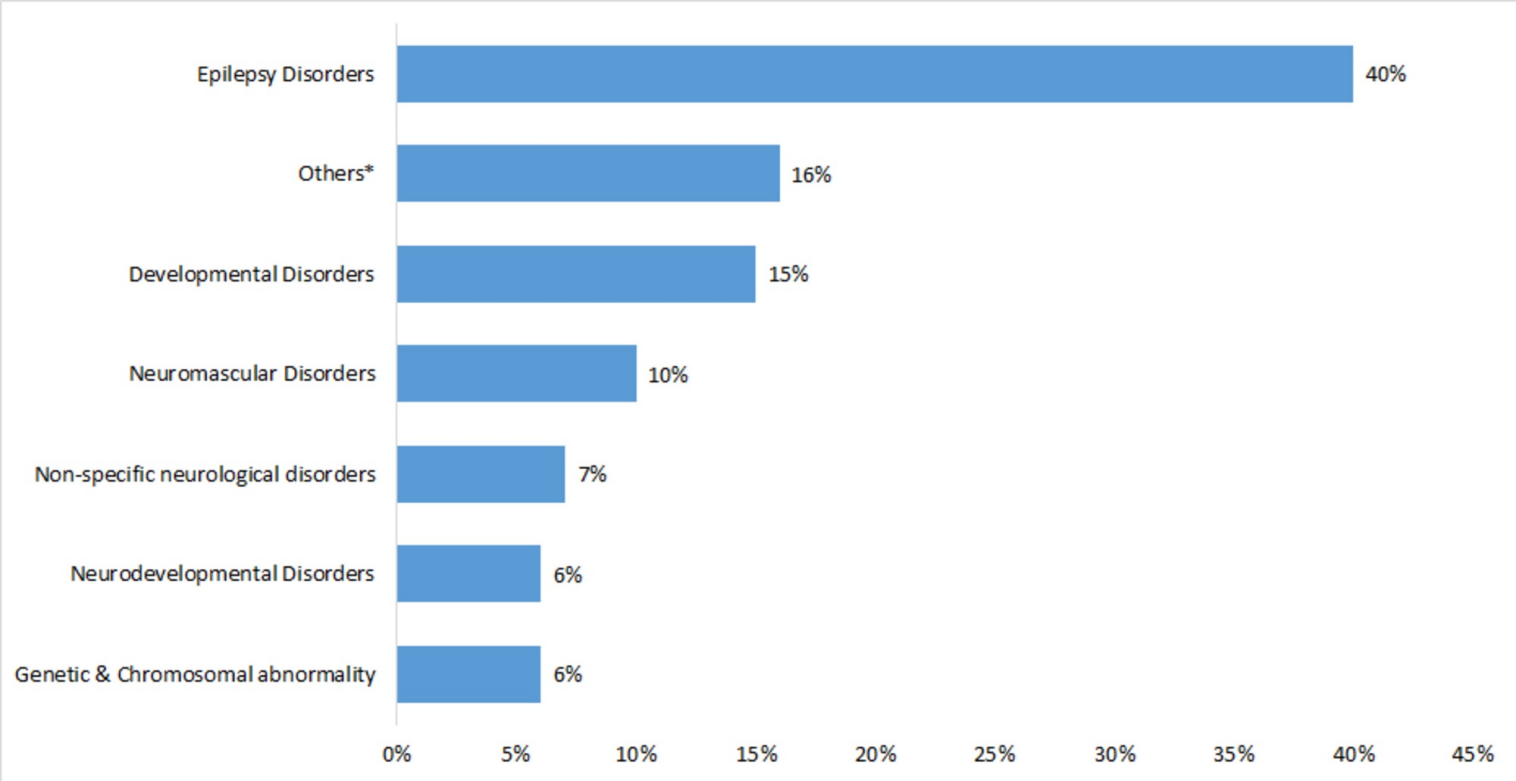
Distribution of neurological diseases among participants (n = 352) *Others include: Infectious & immunological disorders, neurovascular disorders, headaches, congenital disorders, metabolic disorders, neurobehavioral disorders, masses and tumors.

In this study, most of the children suffered from epilepsy disorders (40%, n = 141). Different diseases and disorders (16%, n = 56) were grouped in others because of the small number of children having these diseases such as infectious, immunological disorders, neurovascular disorders, headaches, congenital disorders, metabolic disorders, neurobehavioral disorders, masses and tumors. Developmental disorders were also common (15%, n = 53). To a lesser extent, some children had neuromuscular disorders (10%, n = 35), a non-specific neurological disorders (7%, n = 25), neurodevelopmental disorders (6%, n = 22), genetic and chromosomal abnormalities (6%, n = 20) (Figure 1). Most participants have agreed that they observed side effects of the prescribed medication by the doctor (61%). However, the majority of them (71%) agreed that their child’s condition improved with the prescribed medications. In addition, almost all legal guardians keep their child’s follow-ups with the doctor (99%).

**Figure 2:**
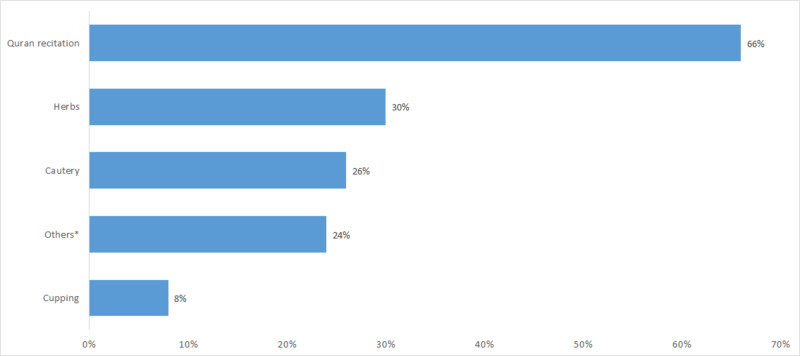
Types of complementary medicine used by participants (n = 227) *Others include exercises, and mixture of different types of CM.

Out of 351 participants (one participant did not answer), the prevalence of using complementary medicine (CM) among the study participants is 42% (n = 148). Moreover, the percentage of people among those who used CM and discontinued after a while is 73% (n = 108). And, only 30% (n = 45) of those who ever used CM do not recommend other people to consume it. The type of CM that was used the most by participants was Quran recitation (66%) followed by herbs (30%), cautery (26%), others (24%) (others include exercises, and mixture of different types of CM), and cupping 8%. Types of CM are described in Figure 2. Most participants agreed that they do not mind telling their doctor about using CM (83%). Many legal guardians observed side effects of CM on their child, while 38% participants noticed an improvement with the CM. Furthermore, only 9% would always visit the traditional healer, and 27% used the CM regularly.

Regarding the time when the parents started giving their children CM, the results show that 45% (n = 66) of parents who consumed CM (n = 148) used it for their child before they see a doctor, while 13% (n = 19) do not remember when they started using it. The top reasons for using CM according to the parents were to alleviate their child’s symptoms (61%, n = 89), followed by to complement the modern medicine (33%, n = 48). Another reason was no improvement with the prescribed medications (16%, n = 23). Other reasons were mentioned by the parents (35%, n = 51) which include not seeing a difference in both methods, not having enough knowledge about their child condition, child’s condition deteriorated after prescribed medications and not trusting modern medicine.

Most participants knew about CM from their families and friends (78%, n = 115), social media (26%, n = 39), the internet (12%, n = 18), books (10%, n = 14), or their doctor (1%, n = 2). Other sources (14%, n = 21) mentioned by the participants include religion and people’s advice. The percentage of those people that never used CM and were advised to use it was 54% (n = 108) and 27% (n = 54) of them thought about using CM at a moment.

Further analysis of the legal guardians' level of education and its relation to the use of CM showed a higher proportion of guardians who have university and above are among those who used complementary medicine (46% fathers, 47% mothers) compared to the guardians that never used CM with the same level of education (32% fathers, 39% mothers). Further analysis of the common complementary treatments used among university-educated fathers and mothers is described in Table 2. The results also showed that fathers and mothers with university education and above commonly used Quran recitation more than any other type of CM. The percentage of university-educated fathers and mothers who used Quran recitation was higher compared to other educational groups (66% for all participants, 79% university-educated fathers, 71% university-educated mothers).

**Table 2:** The relationship between disease categories and complementary medicine use

Demographic Characteristics	Never used CM		Used CM		p-value
	n	%	n	%	
Father's Education					0.049
Illiterate	7	3%	2	1%	
Primary	20	10%	8	5%	
Intermediate	17	8%	14	9%	
Secondary	94	46%	56	38%	
University and above	65	32%	68	46%	
Mother's Education					0.023
Illiterate	20	10%	9	6%	
Primary	19	9%	14	9%	
Intermediate	31	15%	8	5%	
Secondary	54	27%	48	32%	
University and above	78	39%	69	47%	

## Discussion

The prevalence of using complementary medicine (CM) among study participants is relatively high. However, the majority of those who utilized complementary medicine have stopped using it for various reasons, but, interestingly, most of the participants recommended the consumption of complementary medicine which did not meet our expectations. Quran recitation was the most commonly used traditional healing method, especially in above secondary level of educated parents, along with cautery, and herbs (herbs include Anise, herbal oils, and Asafoetida), as well as other modalities of healing that include exercises, aromatherapy and mixture of different types of CM. On the other hand, cupping was not encountered as much in this study, and the reason could be because of the invasive nature of this type of CM.

The demographic data in this study showed a statistical significance between the parents’ educational level and the consumption of CM. It revealed that parents who have higher education were more likely to use CM especially Quran recitation, which was the commonest method used in the study. However, the reason behind this could be attributed to the religious beliefs of the parents rather than their education. Most of the legal guardians in this study are in early to middle adulthood with middle monthly income, and nearly all of the children included in the study have not yet completed their first decade in life. Also, half of the respondents’ origin was in the central region, and the vast majority was living in cities. This may be due to the place that we chose where only children up to fourteen-year-old are accepted and it is a hospital for National Guard workers only. We noticed that age, gender, family income, and origin are not determinants of usage of complementary medicine.

Epilepsy disorders are the most encountered diseases in this study, accompanied by developmental and neuromuscular disorders. Almost every legal guardian claims to show up for the follow-up, however, there is a minority of those who are not compliant with the prescribed medications, and not all of them noticed significant improvement regarding their child’s condition while using the medications. The time of utilization of complementary medicine does not depend on the first visit to the licensed physician. Almost equal percentages of those who ever used alternative medicine started the utilization before and after seeing a doctor. On top of that, many of those complementary medicine users attributed the reason behind starting CM to that it can alleviate their child’s symptoms. Another reason is that they use CM only to complement modern medicine, but almost everyone denies the mistrust of modern medicine. Moreover, common causes of using complementary medicine include religious beliefs and people’s advice.

Most of the participants knew about alternative medicine from family, friends, relatives, and social media, while common sources of knowledge about complementary medicine include religious teaching and the internet. Additionally, the vast majority of legal guardians do not feel embarrassed when telling their child’s physician that they use CM, very few take their child to a traditional therapist, and many of the participants do not use CM commonly since many of them deny any improvement in their child's condition, and lots of them noticed side effects regarding the use of CM. The majority of those who have never tried complementary medicine have thought of using it; we think that is true because many of those who never used complementary medicine were advised by either friends or family.

This study is first to look for the prevalence of complementary medicine usage among children visiting the neurology clinic in Saudi Arabia. Globally, a study done in the United States (US) showed that 34% used CM and another study found that 42% of the US citizens used CM in the past year [13]. These findings are similar to ours regardless of the difference between the two cultures. We found that many parents seek complementary treatment for their children in one way or the another (42%), which is larger to some extent than a local study that was done on families about their children where only 37% of them tried CM. However, this may be due to the nature of our participants with the majority of them having epilepsy disorders which are difficult to understand. Similarly, Quran recitation was the most used CM in both studies [14].

Other studies also were done but most of them were targeting the adult population; nevertheless, that does not prevent us from comparing. Two studies found that 42% of their participants tend to use CM. And in both studies, spiritual healing by the Quran was the commonest CM followed by herbs, cautery, then cupping [12,14]. Surprisingly, their results are identical to ours regarding the prevalence and the most common CM used. Moreover, family and friends were the primary sources of how our population knew about CM which is also similar to both studies [12,14]. This resemblance may stem from the fact that all of these studies including ours were done in Riyadh and maybe because the parents in our study who are the adults in the others.

However, some other studies show a higher percentage of CM consumption. For instance, two studies in Saudi Arabia concluded that 85% and 90.5% of their participants used CM which is double of our findings, but it could be because they considered prayer and supplication as a form of CM, and this could be a form of bias because praying and supplicating for a sick person is a strong belief in Islam [15,16]. In another example, 67% of people with chronic, disabling and incurable neurological diseases used CM, and cupping was the most used CM followed by herbs, cautery, and then Quran. The higher percentage may be due to the unavailability of medical treatment. Also, we could see that cupping was the most used which is opposite to our findings. This could be attributed to the debilitating nature of their illness [7].

One of the limitations is that this study was a questionnaire-based study, and participants had full authority to answer the questions. Therefore, some of the questions were left unanswered or answered incorrectly due to the differences in understanding and interpretations. Moreover, this study was done in one clinic, and in one center which may not represent the opinion of the whole community. For the future, it is crucial to implement similar studies worldwide, not only on pediatrics but on the whole population.

## Conclusions

Almost half of the parents in this study have used complementary medicine (CM) on their children, especially Quran recitation. While some parents did not utilize it, the majority of them had been advised/thought to use it. Therefore, we believe that this type of literature would lead to more recognition, not only by the physicians, but also by families of the patients about the trends of utilizing CM in our society, and in turn, should lead to safer practice and trusted communication between the two parties.
